# Supplementary material for: High Biodiversity Arises from the Analyses of Morphometric, Biochemical and Genetic Data in Ancient Olive Trees of South of Italy
Source: Plants (Basel). 2019 Aug 22;8(9):297. doi: 10.3390/plants8090297 (PMC6783963; doi:10.3390/plants8090297)
Supplement: Supplementary file 1 [file plants-08-00297-s001.pdf]

Supplementary material

1. Supplementary tables

Table S1. Olive tree samples generic information.

Sample_ID	Label	Province	Municipality	UTM Est [m]	UTM Nord [m]	MASL [m]	Estimated age [y]
AUL1	CE	SA	Auletta	534267.413	4492947.504	372	132
AUL2	CE	SA	Auletta	534264.370	4492946.713	372	203
AUL3	CE	SA	Auletta	534430.350	4491933.233	346	209
AUL4	CE	SA	Auletta	534446.498	4491955.508	351	255
AUL5	CE	SA	Auletta	534715.368	4491760.944	315	118
AUL6	CE	SA	Auletta	534868.400	4491583.060	293	126
AUL7	CE	SA	Auletta	535497.580	4491437.240	291	191
AUL8	CE	SA	Auletta	535539.750	4491087.730	281	338
AUL9	CE	SA	Auletta	536713.234	4490091.352	310	357
AUL10	CE	SA	Auletta	536793.104	4490152.798	324	128
AUL11	CE	SA	Auletta	537281.800	4490567.494	367	114
AUL12	CE	SA	Auletta	537330.658	4490564.964	325	303
AUL13	CE	SA	Auletta	536570.543	4488515.521	298	209
AUL14	CE	SA	Auletta	536566.010	4488489.268	297	215
AUL15	CE	SA	Auletta	536231.947	4488509.527	246	154
AUL16	CE	SA	Auletta	536282.429	4488478.995	248	194
BUC1	CE	SA	Buccino	532727.802	4495565.835	207	187
BUC3	CE	SA	Buccino	532150.142	4495122.742	188	113
BUC4	CE	SA	Buccino	532126.319	4495094.888	188	230
BUC5	CE	SA	Buccino	529401.035	4493959.384	164	133
BUC6	CE	SA	Buccino	529403.429	4493953.066	163	186
BUC7	CE	SA	Buccino	526891.419	4495352.099	186	134
BUC8	CE	SA	Buccino	526953.777	4495349.660	191	116
BUC10	CE	SA	Buccino	528045.836	4494659.587	156	153
CAG1	CE	SA	Caggiano	538327.991	4490677.031	466	203
CAG2	CE	SA	Caggiano	538296.130	4490749.131	467	117
CAG3	CE	SA	Caggiano	538800.987	4490376.989	466	120
CAG4	CE	SA	Caggiano	538802.953	4490357.129	466	192
CAG5	CE	SA	Caggiano	540274.390	4490086.200	525	180
CAG6	CE	SA	Caggiano	540391.997	4490133.454	536	133
CAG7	CE	SA	Caggiano	540447.444	4490087.577	541	476
CAG8	CE	SA	Caggiano	540483.849	4490087.330	542	102
CAG9	CE	SA	Caggiano	539534.417	4489697.274	435	133
CAG10	CE	SA	Caggiano	539535.744	4489734.467	440	304
PER1	CE	SA	Pertosa	538213.492	4488272.977	327	369
PER2	CE	SA	Pertosa	538209.200	4488366.976	328	104
PER3	CE	SA	Pertosa	538227.354	4488343.980	332	236
PER4	CE	SA	Pertosa	537454.032	4488534.871	244	119
PER5	CE	SA	Pertosa	537464.207	4488532.036	244	136
PER6	CE	SA	Pertosa	537368.092	4488719.373	244	166

PER7	CE	SA	Pertosa	537374.431	4488670.896	235	211
PER8	CE	SA	Pertosa	537386.547	4488652.530	231	112
PER9	CE	SA	Pertosa	537292.681	4488712.335	252	238
PER10	CE	SA	Pertosa	537300.273	4488752.002	255	129
PET1	CE	SA	Petina	533537.035	4490079.060	288	120
PET2	CE	SA	Petina	533513.788	4490090.277	286	203
PET3	CE	SA	Petina	533529.536	4489581.397	307	112
PET4	CE	SA	Petina	533509.645	4489580.197	313	230
PET5	CE	SA	Petina	533490.844	4489562.574	318	127
PET6	CE	SA	Petina	533481.070	4489533.114	327	250
PET7	CE	SA	Petina	533508.200	4489506.151	326	229
PET7BIS	CE	SA	Petina	533519.576	4489536.839	317	257
PET8	CE	SA	Petina	533488.676	4489536.257	323	162
SALV1	CE	SA	Salvitelle	538445.711	4495143.411	236	198
SALV2	CE	SA	Salvitelle	538469.812	4495167.512	241	244
SALV3	CE	SA	Salvitelle	539846.111	4494049.211	268	113
SALV4	CE	SA	Salvitelle	539846.111	4494049.211	268	118
SALV5	CE	SA	Salvitelle	537690.265	4493830.312	450	120
SALV6	CE	SA	Salvitelle	537707.789	4493828.624	452	123
SALV7	CE	SA	Salvitelle	538097.087	4493602.378	529	123
SALV11	CE	SA	Salvitelle	538510.023	4493293.243	565	126
SALV12	CE	SA	Salvitelle	538547.831	4493346.055	569	260
A1	CM	SA	Ascea	515148.000	4444091.000	163	331
A2	CM	SA	Ascea	515123.000	4444066.000	152	405
A3	CM	SA	Ascea	515376.000	4443680.000	167	365
A4	CM	SA	Ascea	515367.000	4443693.000	166	264
A5	CM	SA	Ascea	515488.000	4443490.000	170	388
A6	CM	SA	Ascea	515477.000	4443472.000	168	291
A7	CM	SA	Ascea	515481.000	4443395.000	176	432
A8	CM	SA	Ascea	515456.000	4443391.000	175	462
A9	CM	SA	Ascea	515724.000	4443432.000	212	318
C6	CM	SA	Ceraso	522578.000	4450585.000	485	314
CT1	CM	SA	Centola	523723.000	4436764.000	162	351
CT2	CM	SA	Centola	523726.000	4436783.000	164	324
CT3	CM	SA	Centola	523294.000	4436260.000	59	381
CT4	CM	SA	Centola	523271.000	4436269.000	52	378
CT5	CM	SA	Centola	525226.000	4433238.000	19	361
CT6	CM	SA	Centola	525163.000	4433227.000	17	334
CT8	CM	SA	Centola	525838.000	4433935.000	166	291
CT9	CM	SA	Centola	526663.000	4435437.000	297	351
CT10	CM	SA	Centola	526639.000	4435445.000	290	331
P1	CM	SA	Pisciotta	517241.000	4441864.000	178	388
P2	CM	SA	Pisciotta	517335.000	4441857.000	170	338
P3	CM	SA	Pisciotta	518276.000	4441432.000	191	341
P4	CM	SA	Pisciotta	518896.000	4440628.000	189	274
P5	CM	SA	Pisciotta	518910.000	4440646.000	191	277

P6	CM	SA	Pisciotta	519699.000	4439475.000	95	486
P7	CM	SA	Pisciotta	519832.000	4439485.000	105	654
P8	CM	SA	Pisciotta	519879.000	4439468.000	89	697
P9	CM	SA	Pisciotta	519814.000	4439476.000	101	489
SM1	CM	SA	San Mauro La Bruca	524295.000	4437940.000	236	304
SM2	CM	SA	San Mauro La Bruca	524191.000	4438266.000	234	307
SM3	CM	SA	San Mauro La Bruca	524188.000	4438268.000	234	324
SM4	CM	SA	San Mauro La Bruca	524339.000	4438416.000	235	509
SM5	CM	SA	San Mauro La Bruca	524407.000	4438411.000	238	266
OIR1	OIR	SA	Fisciano	481145.468	4513652.569	213	245
OIR2	OIR	SA	Fisciano	479992.490	4514543.620	422	300
OIR3	OIR	SA	Fisciano	479984.800	4514532.140	186	289
OIR4	OIR	SA	Fisciano	479988.590	4514511.790	186	318
OIR8	OIR	SA	Bracigliano	475599.046	4519354.108	359	360
OIR9	OIR	SA	Bracigliano	475599.548	4519327.242	361	379
OIR15	OIR	AV	Chiusano di San Domenico	494233.166	4534038.513	610	271
OIR16	OIR	AV	Lapio	493792.222	4535382.667	566	240
OIR17	OIR	AV	Lapio	493773.380	4535394.007	572	304
OIR18	OIR	AV	Lapio	494641.513	4536391.519	514	252
OIR19	OIR	AV	Lapio	494608.816	4536432.065	502	230
OIR23	OIR	AV	Lapio	495859.794	4536565.902	492	217
OIR24	OIR	AV	Lapio	495804.855	4536563.607	497	234
OIR25	OIR	AV	Lapio	495707.005	4537413.330	476	184
OIR26	OIR	AV	Lapio	495544.882	4537225.369	530	243
OIR28	OIR	AV	Lapio	495474.665	4537261.831	496	219
OIR29	OIR	AV	Lapio	495488.284	4537246.274	508	248
OIR30	OIR	AV	Taurasi	496991.934	4539562.162	401	218
OIR31	OIR	AV	Taurasi	497094.771	4539543.586	408	213
OIR32	OIR	AV	Sant'Angelo all'Esca	499315.962	4538337.143	454	209
OIR33	OIR	AV	Lugosano	499466.310	4537835.477	460	234
OIR34	OIR	AV	Lugosano	499539.150	4537801.398	451	219
OIR35	OIR	AV	Lugosano	499531.666	4537853.347	462	286
OIR36	OIR	AV	Sant'Angelo all'Esca	498891.645	4539445.628	390	250
OIR38	OIR	AV	Sant'Angelo all'Esca	498858.940	4539495.366	392	292
OIR40	OIR	AV	Sant'Angelo all'Esca	498993.039	4539905.862	399	299
OIR41	OIR	AV	Mirabella Eclano	499319.683	4542209.278	364	365
OIR42	OIR	AV	Mirabella Eclano	499307.920	4542249.904	362	353
OIR43	OIR	AV	Mirabella Eclano	499708.224	4542295.731	413	259
OIR44	OIR	AV	Mirabella Eclano	499720.434	4542267.658	424	198
OIR45	OIR	AV	Mirabella Eclano	499730.247	4542238.338	425	276

OIR46	OIR	AV	Bonito	499622.690	4547963.047	442	240
OIR47	OIR	AV	Bonito	499620.507	4547958.162	446	203
OIR50	OIR	AV	Bonito	498423.089	4548108.074	432	402
OIR51	OIR	AV	Bonito	498469.463	4548137.588	443	346
OIR53	OIR	AV	Bonito	498495.250	4548165.116	455	262
OIR56	OIR	AV	Melito Irpino	501637.433	4548649.937	461	227
OIR57	OIR	AV	Melito Irpino	501599.813	4548616.068	474	250
OIR58	OIR	AV	Melito Irpino	501662.963	4548664.263	471	285
OIR61	OIR	AV	Melito Irpino	501719.580	4548622.860	459	331
OIR62	OIR	AV	Melito Irpino	501688.421	4548626.630	451	320
OIR67	OIR	AV	Grottaminarda	505174.086	4546494.878	404	125
OIR68	OIR	AV	Grottaminarda	505154.557	4546508.328	405	196
OIR69	OIR	AV	Grottaminarda	505130.035	4546511.298	406	189
OIR70	OIR	AV	Grottaminarda	505114.009	4546519.763	407	166
OIR72	OIR	AV	Grottaminarda	505125.568	4546453.958	402	128
OIR73	OIR	SA	Fisciano	484388.533	4511846.879	443	199
OIR74	OIR	SA	Fisciano	484418.576	4511861.308	437	207
OSE1	OSE	SA	Contursi Terme	520757.795	4499601.219	256	203
OSE2	OSE	SA	Palomonte	523410.415	4498753.479	267	230
OSE5	OSE	SA	Contursi Terme	522474.797	4499750.626	243	289
OSE6	OSE	SA	Contursi Terme	521834.712	4499540.143	217	282
OSE7	OSE	SA	Contursi Terme	521835.141	4499595.535	229	366
OSE10	OSE	AV	Oliveto Citra	519350.058	4504207.085	389	324
OSE11	OSE	AV	Oliveto Citra	519344.176	4504210.855	365	264
OSE12	OSE	AV	Oliveto Citra	519472.355	4505806.091	207	244
OSE15	OSE	AV	Oliveto Citra	519584.318	4505827.255	197	221
OSE16	OSE	AV	Oliveto Citra	520033.547	4507182.935	191	216
OSE17	OSE	AV	Oliveto Citra	520033.166	4507189.550	177	193
OSE18	OSE	AV	Calabritto	519573.264	4509823.184	257	297
OSE19	OSE	AV	Calabritto	519798.523	4510236.945	244	339
OSE20	OSE	AV	Calabritto	519786.420	4510248.346	244	197
OSE22	OSE	AV	Calabritto	519758.663	4510747.574	272	197
OSE23	OSE	AV	Calabritto	519771.273	4510735.842	263	183
OSE24	OSE	AV	Calabritto	519376.470	4513398.270	330	230
OSE25	OSE	AV	Calabritto	519375.582	4513382.727	342	247
OSE26	OSE	AV	Calabritto	519319.618	4513937.167	393	241
OSE27	OSE	AV	Calabritto	519323.598	4513938.432	384	247
OSE29	OSE	AV	Calabritto	520398.898	4517187.444	346	270
OSE31	OSE	AV	Caposele	519091.557	4517846.997	400	254
OSE32	OSE	AV	Caposele	519116.592	4517852.390	401	256
OSE34	OSE	AV	Caposele	519494.454	4517175.791	396	225
OSE36	OSE	AV	Caposele	519556.300	4517169.072	377	254
OSE37	OSE	AV	Caposele	519255.934	4516714.711	441	231

Table S2. Shapiro – Wilk test (W statistic) and Levene test (F statistic) results for morphometric variables. The rejection of the null hypothesis of normal data distribution (for the Shapiro – Wilk test) and homoschedasticity of variances (for the Levene test) occurs for a p-value < 0.05. Significance levels: 0.05 (*), 0.01 (**), 0.001 (***).

Morphometric parameters	Shapiro – Wilk test (W)				Levene test (F)
	Collection site				
	CE	CM	OIR	OSE	
Olive weight	0.96 *	0.91 *	0.98	0.94	4.95 **
Major olive axis	0.94 **	0.96	0.98	0.95	1.11
Leaf area	0.96	0.95	0.96	0.94	1.93

Table S3. Shapiro – Wilk test (W statistic) and Levene test (F statistic) results for fatty acids variables. The rejection of the null hypothesis of normal data distribution (for the Shapiro – Wilk test) and homoschedasticity of variances (for the Levene test) occurs for a p-value < 0.05. Significance levels: 0.05 (*), 0.01 (**), 0.001 (***).

Fatty acids	Shapiro – Wilk test (W)				Levene test (F)
	Collection site				
	CE	CM	OIR	OSE	
Palmitic	0.97	0.94	0.91	0.98	11.07 ***
Palmitoleic	0.94 **	0.86 ***	0.65 ***	0.93	7.22 ***
Heptadecenoic	-	0.97	0.32 ***	0.36 ***	10.53 ***
Oleic	0.98	0.96	0.86 ***	0.97	6.44 ***
t-Octadecenoic	0.97	0.74 ***	0.27 ***	-	2.74 *
Linoleic	0.98	0.97	0.82 ***	0.94	9.93 ***
Linolenic	0.94 **	0.95	0.77 **	0.96	4.51 **
Eicosanoic	0.98	0.93 *	0.21 ***	-	16.50 ***
Eicosenoic	-	-	0.70	0.91 *	16.53 ***
Behenic	-	0.88 **	0.21 ***	0.41 ***	10.93 ***

Table S4. Results of Kruskal-Wallis test for the analysis of variance of morphometric variables and concentration of fatty acids. The value of the χ^2 statistic resulted in highly significant (p-value < 0.001, ***) for every variable.

**Variable**	**χ^2 statistic**
Morphometric parameters	
Olive weight	84.5
Major olive axis	36.3
Leaf area	51.7
Fatty acids	
Palmitic	84.1
Palmitoleic	106.7
Heptadecenoic	123.6
Oleic	84.6
t-Octadecenoic	137.7
Linoleic	41.2
Linolenic	91.8
Eicosanoic	142.9
Eicosenoic	139
Behenic	137.8

Table S5. List of samples present in each of the eight genotypes that contains at least four clones (in the same order as they are represented in the dendrogram in Figure 2).

Collection site	Genotype	Ramets
OSE	1	OSE37, OSE27, OSE10, OSE26
CE	2	SALV6, SALV2, SALV1, PET7BIS, PER9, PER7, PER6, CAG6, BUC10, BUC6, AUL8, AUL4, AUL5
CE	3	PET8, PER5, CAG9, AUL13, CAG1
CE	4	SALV4, PET7, PET5, PER4, CAG10, AUL9, CAG8
OSE, CE	5	OSE36, OSE20, OSE19, SALV1, PET4, PET3, PET2, PER10, CAG2, BUC7, AUL1, AUL3
CE	6	PER3, PER2, PER1, CAG4, BUC4, BUC3, AUL10, AUL6, AUL7
CM	7	SM5, SM4, SM3, SM1, P7, P6, P5, P4, P2, CT9, CT8, CT5, CT3, CT1, A7, A6, A3, A5
OIR	8	OIR36, OIR35, OIR31, OIR33

Table S6. Relative weights (loadings) of every fatty acid on each principal component.

**Fatty acids**	**PC1**	**PC2**
Palmitic	0.314	0.318
Palmitoleic	0.235	-0.449
Heptadecenoic	0.224	0.471
Oleic	-0.405	0
t.Octadecenoic	0.343	-0.374
Linoleic	0.305	0
Linolenic	-0.184	0.326
Eicosanoic	0.425	0
Eicosenoic	-0.408	0.145
Behenic	0.199	0.446

2. Supplementary figures

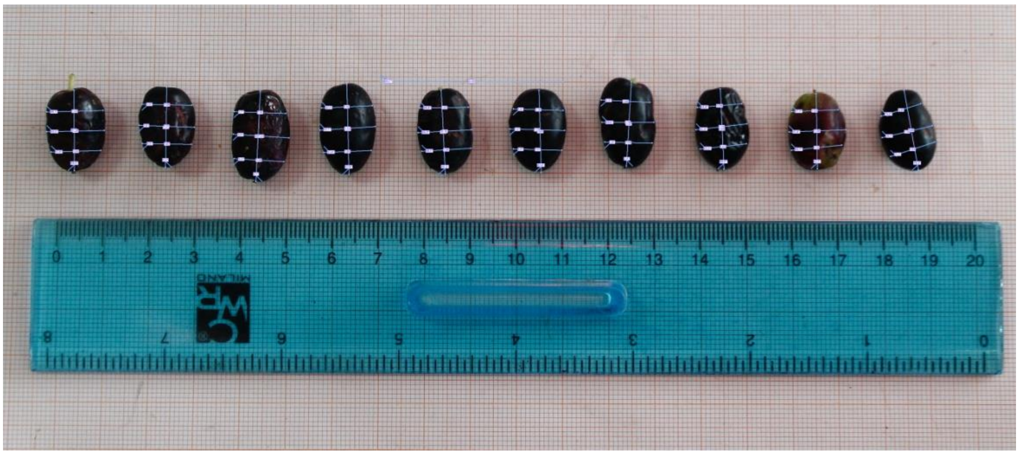

Figure S1. Measurement of the size of the olives carried out on a photographic reference with the Adobe Photoshop CC software.

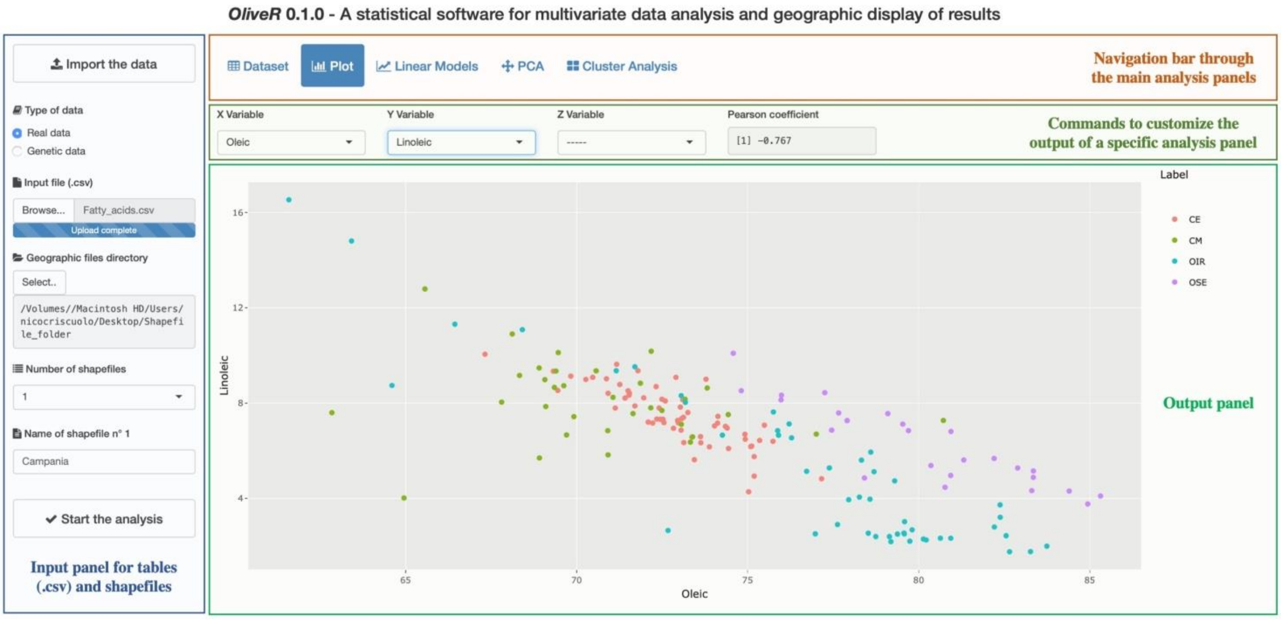

Figure S2. *OliveR* software layout after loading real data and shapefiles. The main panels of the application are highlighted.

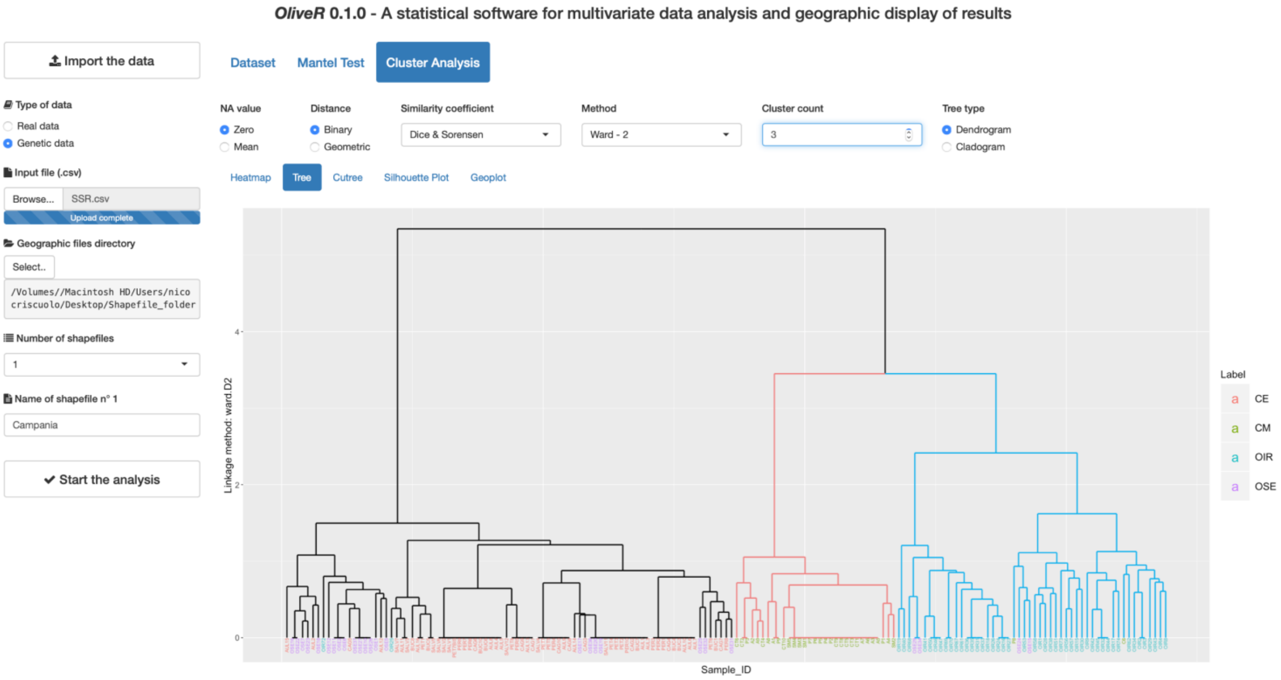

Figure S3. *OliveR* software layout after loading genetic data (loci bp) and shapefiles.

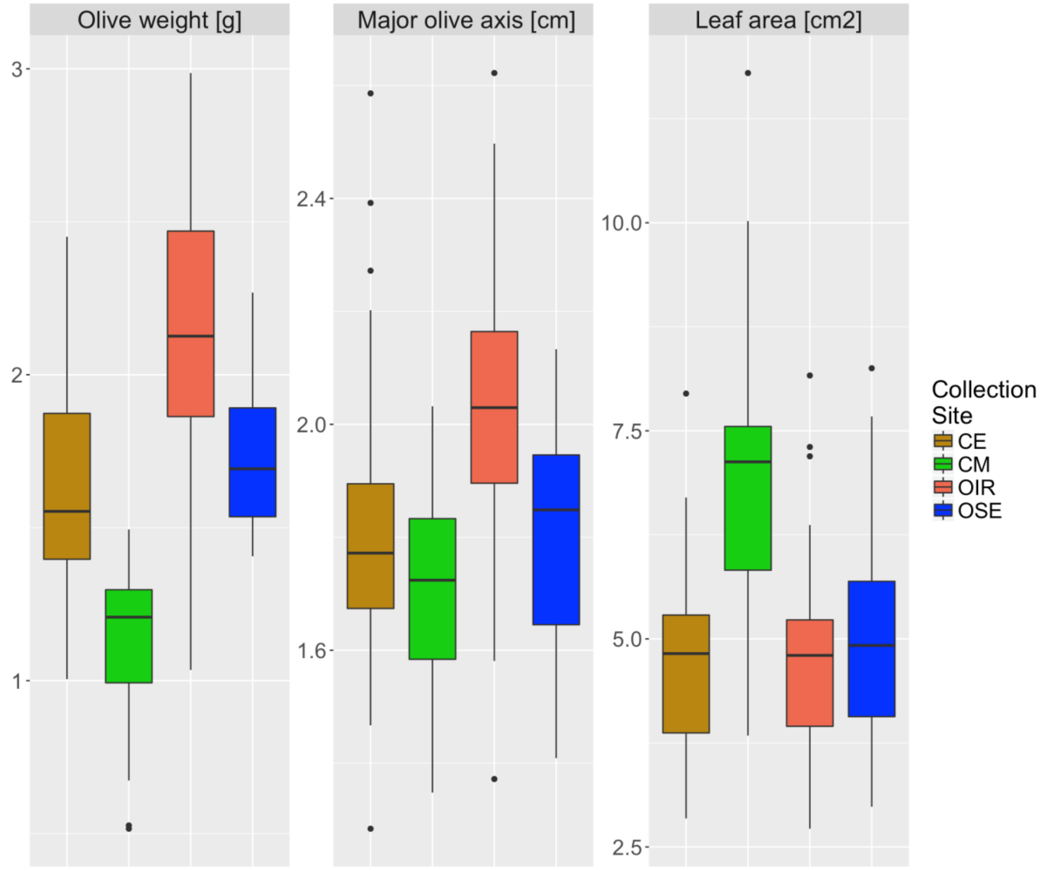

Figure S4. Boxplot of morphometric parameters per collection site.

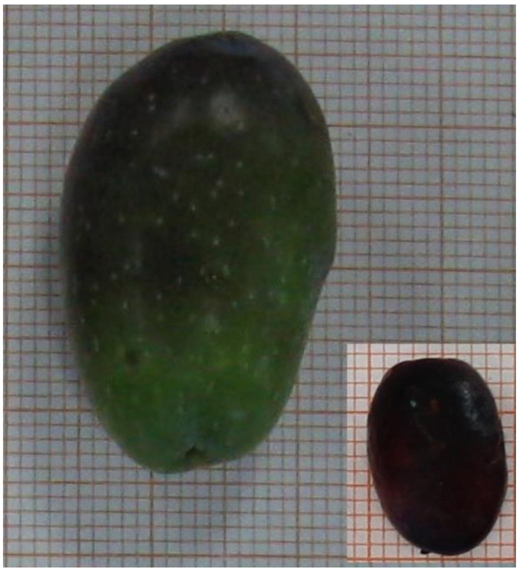

Figure S5. Photographic comparison on graph paper between an olive of the OIR collection site (the largest) and one of the CM collection site.

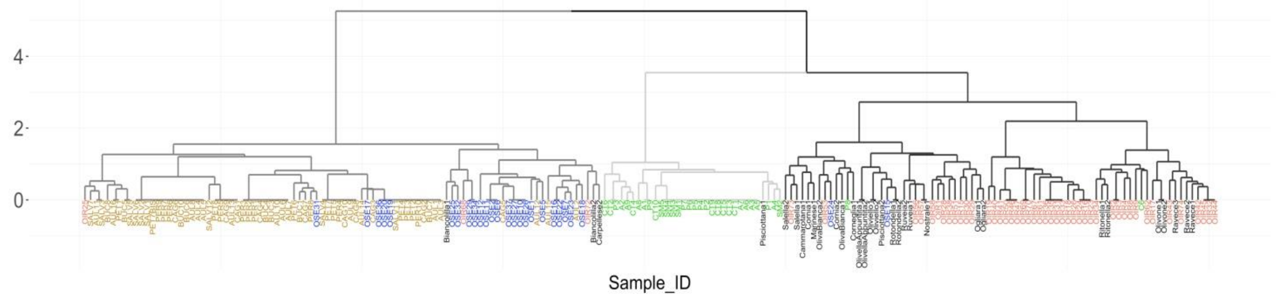

Figure S6. Dendrogram calculated with the Ward method on the genetic distance among samples, based on the similarity index of Dice-Sørensen, and including some known cultivars as reference samples

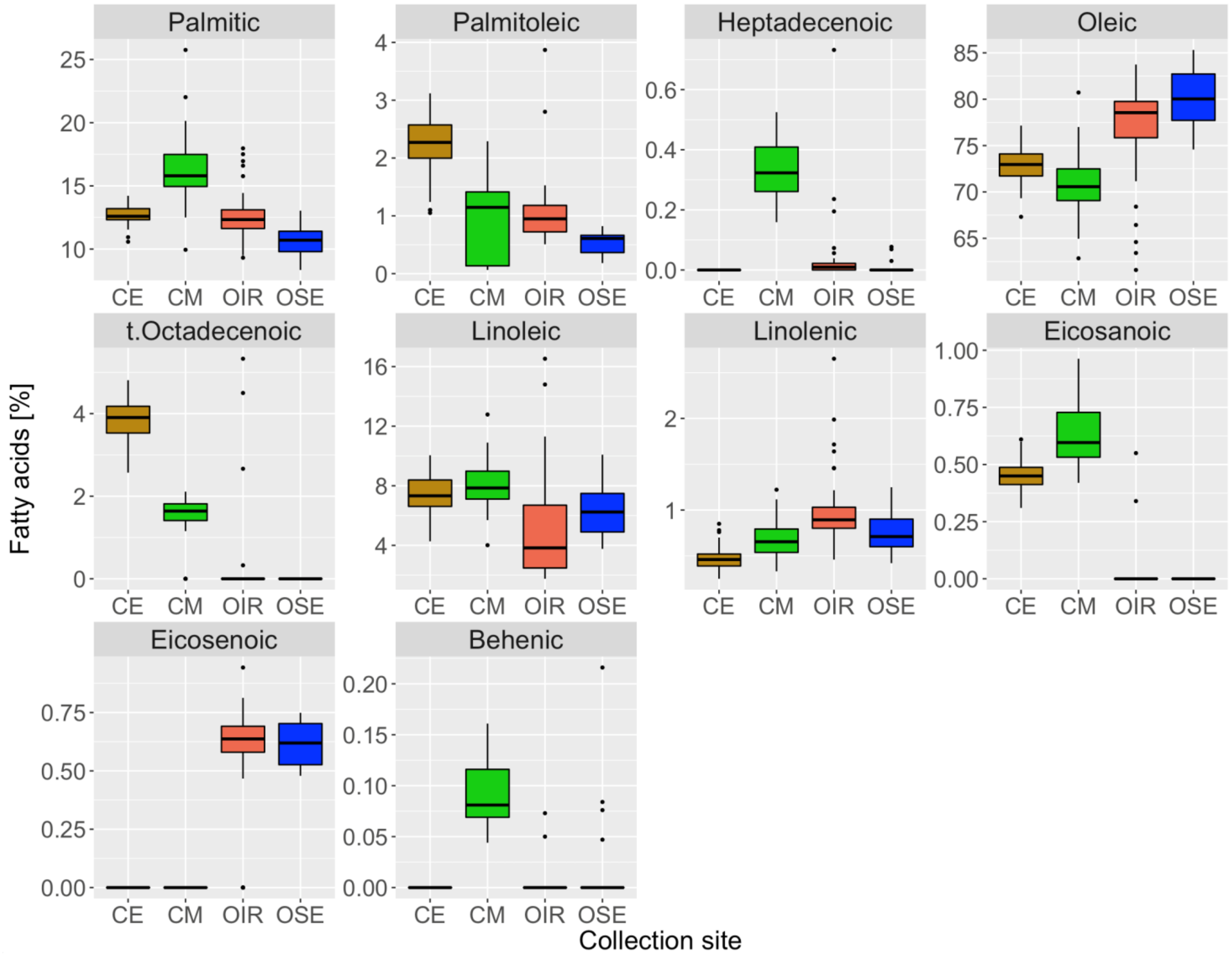

Figure S7. Boxplot of fatty acid concentrations per collection site.

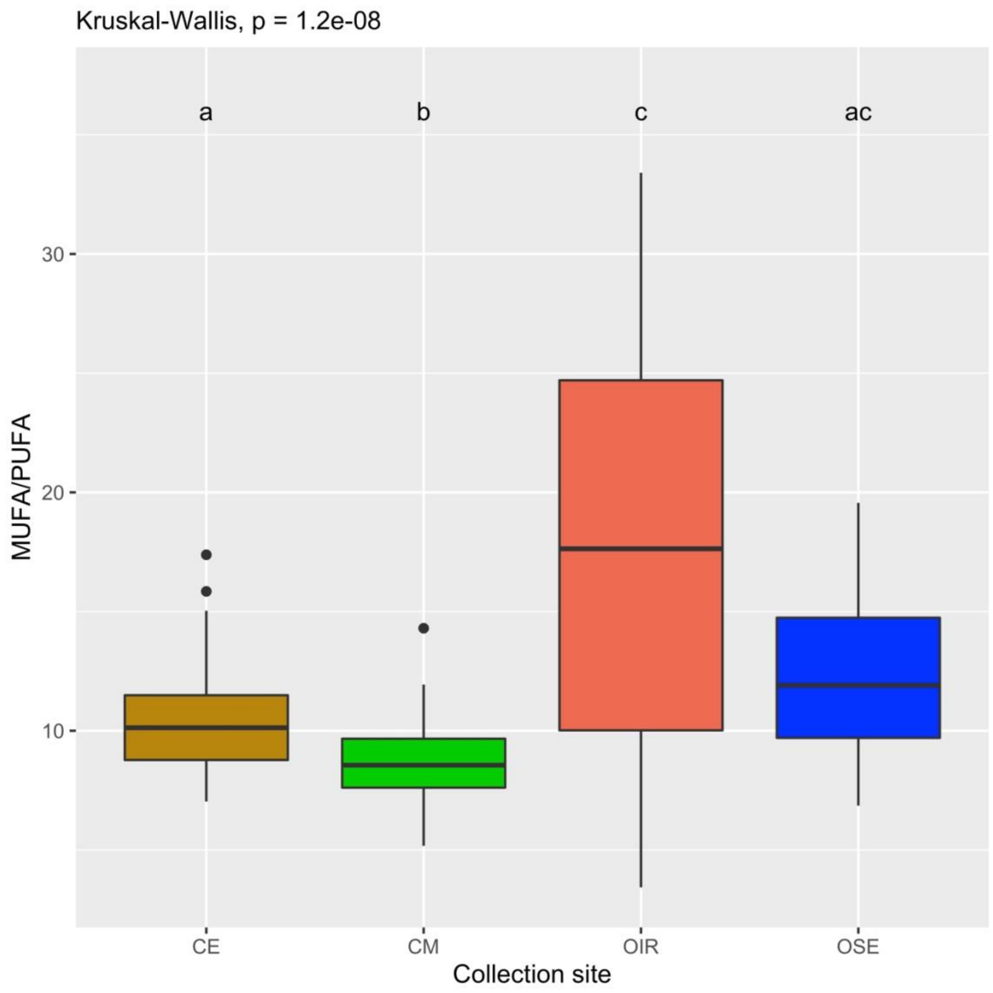

Figure S8. Boxplot of MUFA/PUFA ratio per collection site and post-hoc statistical significance difference ($p < 0.05$) between collection sites.

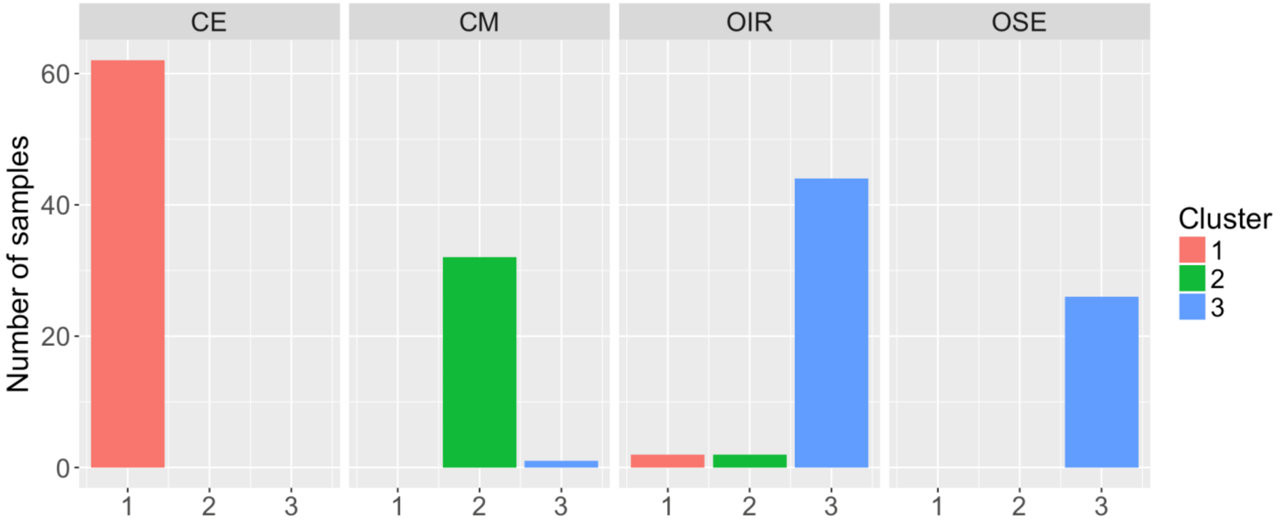

Figure S9. Barplot of the cluster analysis result performed with the PAM method on the first two PCs related to the fatty acid dataset. Clusters are divided by collection sites.

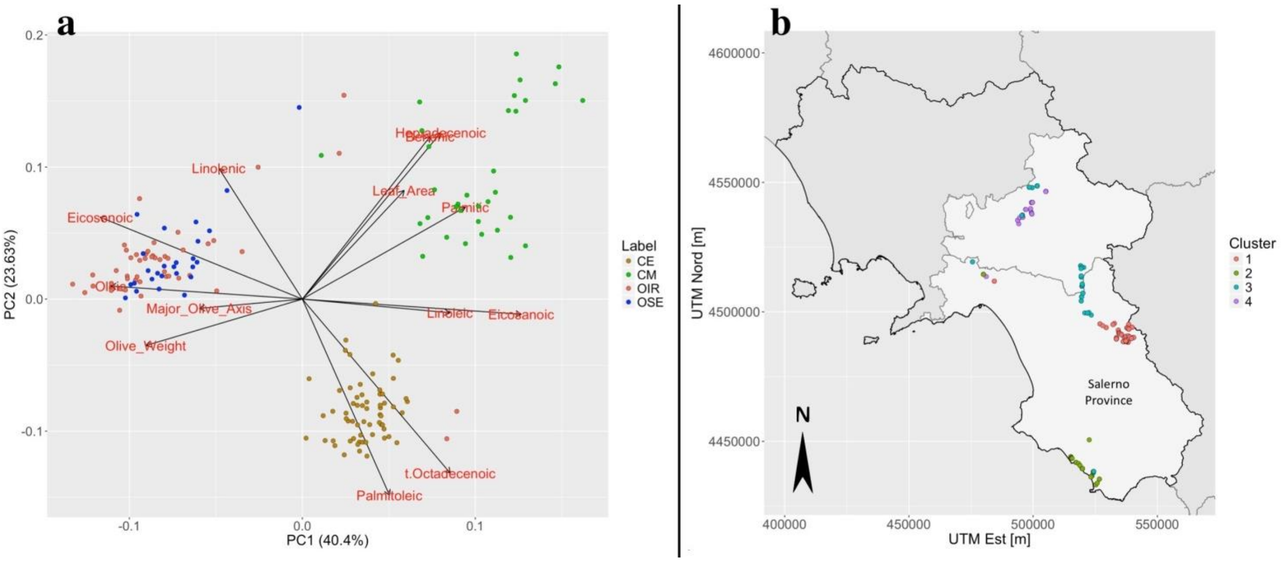

Figure S10. Result of the PCA (a) and subsequent cluster analysis with the PAM method reported on a geographic map (b) performed on the dataset containing the variables of the fatty acids and the morphometric parameters.

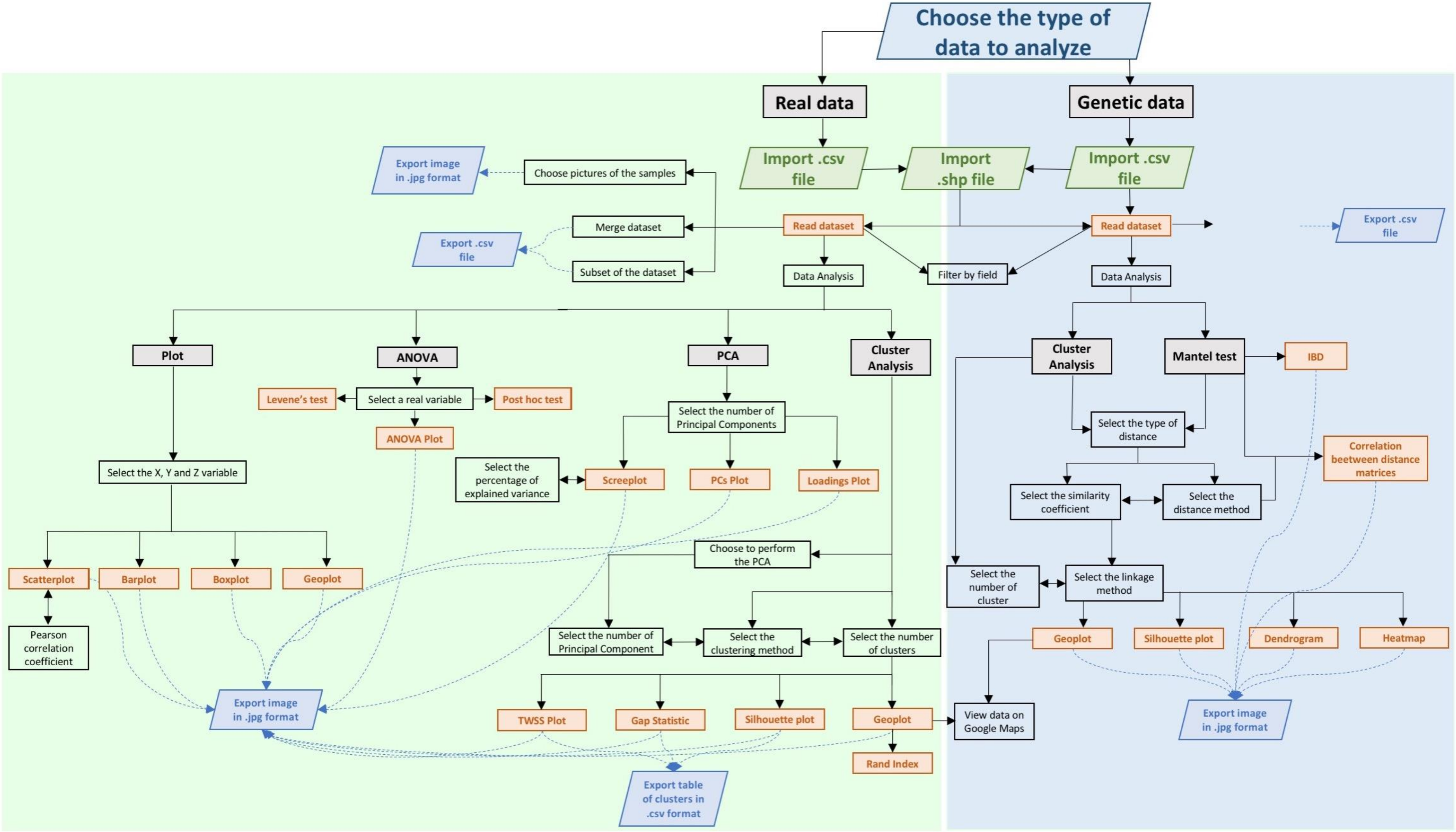

Figure S10. Internal structure of *OliveR* 0.1.0
